# Comparison of ultrashort pulse ablation of gold in air and water by time-resolved experiments

**DOI:** 10.1038/s41377-022-00751-6

**Published:** 2022-03-23

**Authors:** Maximilian Spellauge, Carlos Doñate-Buendía, Stephan Barcikowski, Bilal Gökce, Heinz P. Huber

**Affiliations:** 1 Department of Applied Sciences and Mechatronics, Munich University of Applied Sciences, Lothstraße 34, 80335 Munich, Germany; 2 Technical Chemistry I and Center for Nanointegration Duisburg-Essen (CENIDE), University of Duisburg-Essen, 45141 Essen, Germany; 3 Materials Science and Additive Manufacturing, School of Mechanical Engineering and Safety Engineering, University of Wuppertal, 42119 Wuppertal, Germany

**Keywords:** Laser material processing, Ultrafast photonics, Nanoparticles

## Abstract

Laser ablation in liquids is a highly interdisciplinary method at the intersection of physics and chemistry that offers the unique opportunity to generate surfactant-free and stable nanoparticles from virtually any material. Over the last decades, numerous experimental and computational studies aimed to reveal the transient processes governing laser ablation in liquids. Most experimental studies investigated the involved processes on timescales ranging from nanoseconds to microseconds. However, the ablation dynamics occurring on a sub-nanosecond timescale are of fundamental importance, as the conditions under which nanoparticles are generated are established within this timeframe. Furthermore, experimental investigations of the early timescales are required to test computational predictions. We visualize the complete spatiotemporal picosecond laser-induced ablation dynamics of gold immersed in air and water using ultrafast pump-probe microscopy. Transient reflectivity measurements reveal that the water confinement layer significantly influences the ablation dynamics on the entire investigated timescale from picoseconds to microseconds. The influence of the water confinement layer includes the electron injection and subsequent formation of a dense plasma on a picosecond timescale, the confinement of ablation products within hundreds of picoseconds, and the generation of a cavitation bubble on a nanosecond timescale. Moreover, we are able to locate the temporal appearance of secondary nanoparticles at about 600 ps after pulse impact. The results support computational predictions and provide valuable insight into the early-stage ablation dynamics governing laser ablation in liquids.

## Abstract (web summary)

Ultrafast dynamics of laser ablation of gold in water revealed by pump-probe microscopy on a picosecond to microsecond timescale.

## Graphical abstract

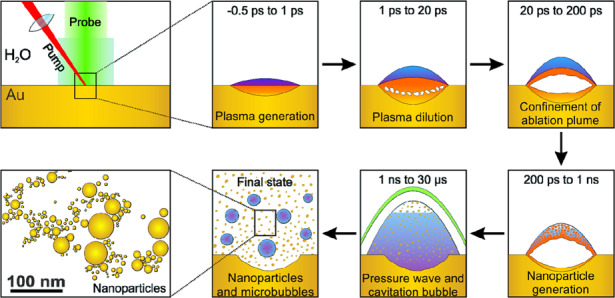

## Introduction

Laser ablation in liquids (LAL) denotes the deposition of laser energy to ablate a material that is covered by a liquid-confinement layer. Since its development in the 1990s^1^, LAL has been effectively leveraged to synthesize colloids^2^, linking the fields of photonics and nanotechnology. In contrast to conventional chemical colloid processing routes, LAL allows for the environmentally friendly generation of surfactant-free nanoparticles (NPs) and offers the unique opportunity of transferability to alloy colloid production^1^. This opens up various applications for NPs produced with LAL, including catalysis^3^, 3D printing^4^, antibacterial nanomaterials^5^, surface-enhanced Raman spectroscopy^6^, photovoltaics^7^, plasmonics^8^, and nanomedicine^9^. Despite the research carried out to maximize the production and control the properties of the generated nanomaterials by LAL, the underlying dynamical processes are not fully understood. The variety of involved physical and chemical processes, as well as highly non-equilibrium dynamics, make the investigation of the transient processes challenging^10^.

Furthermore, in contrast to ablation in gaseous environments, the liquid layer present in LAL adds complexity since it promotes energy loss^11^, represents a highly reactive environment^10^, and confines the ablation products^12^. So far, a large body of research performed in the field of LAL has focused on investigating the transient dynamics occurring on the nanosecond and microsecond timescale. This includes the investigation of the plasma plume composition and dynamics^10^, monitoring of cavitation bubble expansion and collapse^13,14^, and ejection of NPs into the liquid-confinement layer^15,16^. However, investigating the sub-ns dynamics is crucial, as the physical and chemical conditions under which NPs are generated are determined at this timescale^10^.

Molecular dynamics studies of picosecond laser ablation of Ag^16^ and Au^17^ in water identified two mechanisms of NP generation on a timescale of hundreds of picoseconds. Primary NPs with diameters <10 nm condensate from evaporated Ag atoms due to favorable nucleation conditions in a low-density water/metal mixing region. Larger secondary NPs with diameters of a few tens of nm originate from Rayleigh–Taylor and Richtmyer–Meshkov instabilities of a decelerated molten metal layer formed during the early stages of the ablation^16^. Furthermore, nanoparticle/nanodroplet growth and coalescence within the plasma and cavitation bubble is another formation mechanism contributing to the fraction of larger secondary nanoparticles and thus is a key process determining the final colloid^15,18^. Due to spatial scales ranging from nm to mm and temporal scales ranging from fs to µs, experimental testing of computational predictions remains challenging. In recent years, ablation plume confinement, as well as early plasma generation, were observed by pump-probe microscopy^19^. However, an experimental investigation of the complete LAL dynamics ranging from pulse impact on a ps timescale to cavitation bubble formation on a µs timescale is missing up to date.

To address this gap, picosecond time-resolved pump-probe microscopy measurements near the ablation threshold are performed, enabling the investigation of the transient surface reflectivity on a timescale spanning from ps to the µs. The temporal resolution achieved together with the wide time span that can be analyzed in a single measurement allow us to obtain the complete spatiotemporal single-pulse ablation dynamics of gold (Au) in air and water after irradiation with ps pulses. The transient reflectivity after ablation in air, where the ablation mechanisms are well characterized, serves as a reference to analyze the transient reflectivity observed in water and allows to assign characteristic timescales, during which the water confinement layer exerts the largest influence on the ablation dynamics. This way, we are able to draw a concise picture of the ablation dynamics of Au in water and highlight differences compared to ablation in air.

## Results

### Single-pulse ablation threshold fluence of gold in air and water

A direct comparison of the ablation dynamics in air and water requires knowledge of the absorbed peak fluence. However, the dependence of the absorption of the gold target^20^ and the water layer^21^ on the incident peak fluence *Φ*_0_ makes it challenging to determine the exact value of the absorbed peak fluence. In order to compensate for fluence- and ambient medium-dependent absorption, all peak fluences were normalized to the ablation threshold fluence *Φ*_thr_.

Single-pulse ablation experiments in air and water were performed using the pump-pulse (wavelength: 1056 nm; pulse duration: 3 ps) of the pump-probe microscopy setup (see “Materials and Methods” and Supplementary Information, Section 1, Supplementary Fig. S1). The *D*^2^-model yielded ablation threshold fluences of (1.4 ± 0.1) J cm^−2^ and *Φ*_thr_ = (2.1 ± 0.1) J cm^−2^ in air and water, respectively (see Supplementary Information, Section 2, Supplementary Fig. S2a). The determined *Φ*_thr_ agree well with literature values^22^ of 1.5 J cm^−2^ in air and 2.2 J cm^−2^ in water. Furthermore, the model-predicted minor beam waist radii *w*_min_ of (14.6 ± 0.1) µm and (14.4 ± 0.1) µm in air and water, are in agreement with the beam waist radius of *w*_0_ = (15 ± 1) µm, measured in air. Below peak fluences of 1.8 ∙ *Φ*_thr_, ablation with the same multiple of the ablation threshold fluence leads to comparable surface damage in air and water (see Supplementary Information, Section 2 and Supplementary Fig. S2b). This justifies the normalization of the peak fluence to compensate for the fluence-dependent absorption in air and water below 1.8 ∙ *Φ*_thr_. Above 1.8 ∙ *Φ*_thr_ the ablation diameter *D* in water decreases compared to the model predictions, suggesting an additional energy dissipation channel. Since the theoretical determined optical breakdown threshold fluence *Φ*_OB_ = 1.7 ∙ *Φ*_thr_ approximately matches the characteristic peak fluence *Φ*_0_ = 1.8 ∙ *Φ*_thr_, the additional pulse energy dissipation is likely to be a consequence of optical breakdown within the water layer^23^ (see Supplementary Information, Section 3, Supplementary Figs. S3 and S4). This highlights the importance of optical breakdown during ps LAL, where the optical breakdown threshold fluence of a few J cm^−2^ (ref. ^21^) is comparable to peak fluences of several J cm^−2^, typically employed in ps LAL^24^. However, when ns LAL is considered, optical breakdown within the water layer is negligible as the optical breakdown threshold fluences are in the range of several 100 J cm^−2^ (ref. ^21^) and thus far above typical peak fluences of several J cm^−2^ used for the ns LAL process^24^.

To allow for a comparison of *Φ*_thr_ in air and water, reflection losses at the air-gold, air-water, and water-gold interface, as well as linear absorption losses within the water layer, are accounted for^25,26^. This yields absorbed fluences at the ablation threshold of 0.04 J cm^−2^ and 0.06 J cm^−2^ for air and water, respectively. The higher absorbed fluence at the ablation threshold observed in water is unlikely to be caused by additional losses due to optical breakdown since the ablation threshold fluence in water is below the theoretically determined threshold fluence for optical breakdown (*Φ*_OB_ = 1.7 ∙ *Φ*_thr_). The differences between the absorbed ablation threshold fluences may be attributed to different ablation dynamics in air and water. In fact, simulations predict that the water confinement layer decelerates the ablation plume, which results in a re-deposition of ablated material and an obstruction of the ablation process^27^. This may lead to the absence of visible material ablation, even though the ablation threshold fluence is exceeded. Since the *D*^2^-model exclusively considers the final ablated area, the actual ablation threshold fluence determined in water may be overestimated by this model since possible re-deposition is not accounted for.

### Spatiotemporal relative reflectivity change above the ablation threshold

**Fig. 1:**
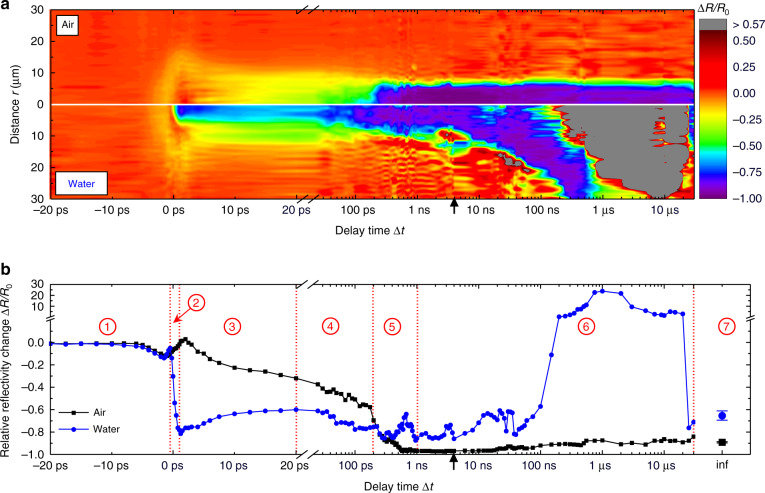
Spatiotemporal reflectivity dynamics in air and water, recorded after subjecting the sample to pump pulses with a peak fluence of 1.5 ∙ *Φ*_thr_. Axis breaks at Δ*t* = 21 ps were introduced in order to highlight the early reflectivity dynamics. **a** Color-coded relative reflectivity change Δ*R*/*R*_0_ as a function of Δ*t* and the distance from the irradiation center *r* for ablation in the air (top half) and water (bottom half). The initial reflectivity of Au in air and water is *R*_0_ = 0.683 and *R*_0_ = 0.636, respectively. Δ*R/R*_0_ = 0.57 corresponds to 100% reflectivity of the Au surface in water. **b** Δ*R*/*R*_0_ averaged over the region *r* ≤ 1.3 µm in (**a**) as a function of Δ*t* in the air (black squares and lines) and water (blue circles and lines). The final state values Δ*R*_inf_/*R*_0_, recorded 5 s after pump-pulse impact are shown at the Δ*t* labeled inf. Seven characteristic temporal domains are separated by red dashed lines and highlighted by red encircled numbers. Black arrows at Δ*t* = 4 ns denote the delay time where the probe-pulse delay is switched from optically to electronically

The transient reflectivity of the Au surface was recorded by the pump-probe microscopy setup described in the materials and methods section in order to investigate the ablation dynamics on a timescale ranging from picoseconds to microseconds Fig. 1 displays the relative reflectivity change Δ*R*/*R*_0_ of the Au surface after the impact of single pump pulses at a wavelength of 1056 nm, a pulse duration of 3 ps and a peak fluence of *Φ*_0_ = 1.5 ∙ *Φ*_thr_. Δ*R*/*R*_0_ was recorded with probe pulses at a wavelength of 528 nm and pulse duration of 500 fs. The initial reflectivity *R*_0_ of Au immersed in air and water is 0.683 and 0.636, respectively^25,26^.

It is evident that the reflectivity dynamics in air and water differ significantly. For the sake of clarity, the variety of observed reflectivity dynamics is divided into seven distinct temporal domains (Fig. 1b).

### Domain 1, from −20 ps to −0.5 ps: laser pulse absorption, heating, and surface melting

In the first temporal domain, which ranges from Δ*t* = −20 ps to Δ*t* = −0.5 ps similar temporal and spatial reflectivity dynamics are observed in air and water. For delay times below Δ*t* = −6 ps, Δ*R*/*R*_0_ remains at 0. Within this time interval, the Δ*R/R*_0_ resolution of approximately 0.005 is visible. When Δ*t* = −6 ps is exceeded, Δ*R*/*R*_0_ decreases to local minima of −0.12 and −0.14 at Δ*t* = −1.5 ps in air and water, respectively. Following the initial reflectivity decrease, the reflectivity recovers from Δ*t* = −1.5 ps onwards and reaches a value of −0.05 (−0.10) at Δ*t* = −0.5 ps in the air (water).

Both, the local reflectivity minimum and the subsequent reflectivity increase in air and water are understood to be a consequence of thermal excitation of electrons by the pump-pulse and subsequent modulated absorption/reflection of the probe pulse as illustrated in previous studies^28,29^.

### Domain 2, from −0.5 ps to 1 ps: plasma formation

**Fig. 2:**
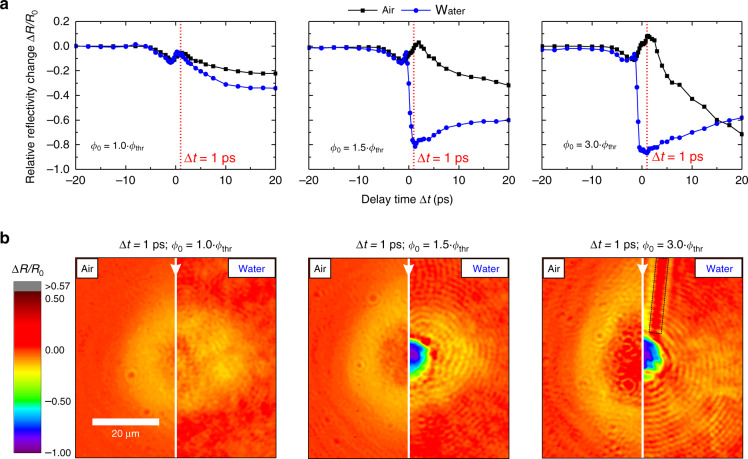
Relative reflectivity change Δ*R*/*R*_0_ of Au immersed in air and water after being subjected to pump pulses with peak fluences of 1.0 ∙ *Φ*_thr_ (left column), 1.5 ∙ Φ_thr_ (middle column), and 3.0 ∙ *Φ*_thr_ (right column). **a** Transient Δ*R*/*R*_0_ for irradiation in air (black lines and squares) and water (blue lines and circles). The red dotted vertical lines indicate a delay time of Δ*t* = 1 ps. **b** Spatially resolved relative reflectivity change at Δ*t* = 1 ps. The vertical white line within each image separates the optical response in air (left half) and water (right half). White arrows indicate the direction from which the pump-pulse was incident on the sample surface. The black dashed rectangle at 3.0 ∙ *Φ*_thr_ highlights the reflectivity change along the pump-pulse propagation path

The reflectivity dynamic in the second domain, which ranges from −0.5 ps to 2 ps is defined by a pronounced reflectivity decrease in water. It is observed that the reflectivity decrease is spatially limited to a region *r* ≤ 6 µm (see Fig. 1a), which reflects the influence of the local fluence on the reflectivity dynamics in domain 2. Hence, the influence of *Φ*_0_ on the fast reflectivity decrease was further investigated. Results obtained after subjecting the sample to single pulses with peak fluences of 1.0 ∙ *Φ*_thr_, 1.5 ∙ *Φ*_thr,_ and 3.0 ∙ *Φ*_thr_ in air and water are depicted in Fig. 2.

From Fig. 2, it is evident that the fast reflectivity decrease in water exhibits a threshold behavior. When Au is irradiated with 1.0 ∙ *Φ*_thr_ the reflectivity dynamics in air and water nearly resemble each other. However, when the sample is irradiated with fluences of 1.5 ∙ *Φ*_thr_ and 3.0 ∙ *Φ*_thr_ a sharp reflectivity decrease with exponential decay times of approximately 0.6 ps and 0.4 ps is present in water around zero delay time. This indicates a nonlinear effect, since the reflectivity decrease proceeds faster than the pump-pulse duration. With a minimum of Δ*R*/*R*_0_ = −0.87, this decrease is more pronounced at 3.0 ∙ *Φ*_thr_, compared to a minimum of Δ*R*/*R*_0_ = −0.78 at 1.5 ∙ *Φ*_thr_. For the incident peak fluence of 1.5 ∙ *Φ*_thr_ the assumption of a Gaussian fluence distribution on the sample surface is justified due to the validity of the *D*^2^-model (see Supplementary Information, Section 2, Supplementary Fig. S2). Here the spatial onset of the sharp reflectivity decrease can be linked to a local fluence which is approximately equal to *Φ*_thr_. When a peak fluence of 3.0 ∙ *Φ*_thr_ is considered, the *D*^2^-model is no longer applicable presumably due to an optical breakdown and thus the assumption of a Gaussian fluence distribution on the sample surface may no longer be valid (see Supplementary Fig. S2). Irradiation of the sample surface at this peak fluence leads to a pronounced reflectivity change along the pump-pulse propagation path (see black dashed rectangle in Fig. 2b, 3.0 ∙ *Φ*_thr_).

To explain the physical origin of the nonlinear reflectivity decrease in water, ultrafast melting, induction of an optical breakdown, and electron emission are considered.

Since the reflectivity decrease in water occurs approximately when the ablation threshold fluence is exceeded, ultrafast melting^30^ of the Au surface may be responsible for the reflectivity decrease. Under the assumption that the Au surface is molten at Δ*t* = 1 ps, respective Δ*R*/*R*_0_ values of −0.12 and −0.17 are calculated for air and water using the optical properties of liquid Au^31^. This estimation yields Δ*R*/*R*_0_ values that indicate a much smaller reflectivity decrease than the one observed in the experiments. Furthermore, it was reported that ultrafast melting occurs already when the melting threshold is slightly exceeded^30^. Since the melting threshold of metals and hence the threshold for ultrafast melting is typically several times lower than the ablation threshold fluence^32^, it is implied that a reflectivity decrease would already be observable for fluences far below the ablation threshold fluence. This contradicts our findings as the fast reflectivity decrease occurs approximately when the ablation threshold fluence is exceeded. Thus ultrafast melting can be eliminated as the sole origin of the nonlinear reflectivity decrease in water.

Another possible explanation for the fast reflectivity decrease in water is the generation of a highly absorbing plasma due to an optical breakdown. Plasmas generated in water can reach electron densities of up to 10^21^ cm^−3^ and exhibit an absorption of ~25% (ref. ^33^). As shown in Section 3, Supplementary Fig. S4 of the Supplementary Information, the calculated optical breakdown threshold fluence in water is exceeded for 3.0 ∙ *Φ*_thr_, while for irradiation with 1.0 ∙ *Φ*_thr_ and 1.5 ∙ *Φ*_thr_ no optical breakdown should occur. In fact, increased absorption of up to Δ*R*/*R*_0_ = −0.30 along the pump-pulse propagation path is only visible after irradiation with 3.0 ∙ *Φ*_thr_ (see Fig. 2b), indicating optical breakdown generation at this peak fluence. However, for 1.5 ∙ *Φ*_thr_ no visible absorption should occur due to optical breakdown. This is contradicted by the observed reflectivity decrease and thus an optical breakdown induced plasma can be excluded to be the sole origin of the high absorption.

Furthermore, a dense electron plasma above the Au target may be generated by thermionic emission of hot electrons^34^. Compared to irradiation in air, higher electron densities are reached in water due to an increased thermionic emission yield (work function of 2.26 eV in water^35^ compared to 4.2 eV in air^36^) and trapping of the electrons by the water layer (electron penetration depth of 10–100 nm in water^19^). The peak electron temperatures at the gold surface were approximately calculated to range from 10 to 20 kK, using the two-temperature model (TTM) (see Supplementary Information, Section 4, Supplementary Fig. S5). Based on this, the resulting electron densities generated by thermionic emission are estimated in a semi-quantitative way. This was done by calculating the total thermionic electron yield in the space-charge limited regime^37^ and assuming an electron penetration depth of 10–100 nm in water^19^, independent of the electron kinetic energy (see Supplementary Information, Section 5). This yields electron densities in the range of 10^15 ^cm^−3^ to 10^16^ cm^−3^ (see Supplementary Information, Section 5, Supplementary Fig. S6). These electron densities lie several orders of magnitudes below the required 10^21^ cm^−3^, thus thermionic emission can be ruled out as the sole mechanism for the fast reflectivity decrease in water. However, it was previously pointed out that multiphoton photoemission rates can exceed thermionic emission rates by three orders of magnitude in the case of tungsten^38^. Assuming the same is true for Au, maximum electron densities in the range of 10^18^ cm^−3^ to 10^19^ cm^−3^ may be reached, which still lie substantially below the required 10^21^ cm^−3^ for significant probe-pulse absorption.

To give a reasonable explanation of the fast reflectivity decrease in water, an interplay of electron emission—by thermionic emission and multiphoton photoemission—and optical breakdown is proposed. The optical breakdown threshold fluence is very sensitive to the number of initial free electrons present in water and decreases with an increasing number of free electrons^39^ (see Supplementary Information, Section 3, Supplementary Fig. S3). Electron emission from the Au surface creates a significant amount of free electrons within the vicinity of the irradiated region. These electrons then may act as a precursor for the optical breakdown, confining the optical breakdown region closely above the irradiated surface. At this point, it should be noted that the electron-emission process occurs as soon as the electron temperature is elevated by the leading edge of the pump-pulse but only becomes visible in the probe-pulse reflectivity after the critical electron density is exceeded by cascade ionization and subsequent optical breakdown.

In conclusion, the proposed explanation satisfies both the observed threshold behavior of the fast reflectivity decrease and the absence of visible absorption along the pump-pulse propagation path for 1.5 ∙ *Φ*_thr_.

### Domain 3, from 1 ps to 20 ps: early mechanical motion and plasma dilution

The optical response in domain 3 is linked to the fluence-dependent plasma generation observed in domain 2 (see Fig. 2a). For a peak fluence of 1.0 ∙ *Φ*_thr_, where no visible plasma generation occurs, Δ*R*/*R*_0_ shows similar transient behaviors in air and water. Here, the reflectivity decreases with comparable exponential decay times of approximately 6 ps. At fluences of 1.5 and 3.0 times above the ablation threshold fluence, the exponential decay time in air increases to a value of 13 ps. In the case of water irradiated above the ablation threshold, the fast reflectivity decrease induced by plasma absorption is followed by an increase of Δ*R*/*R*_0_ to a local maximum of −0.6 at Δ*t* = 20 ps. The increase is characterized by exponential rise times of 7 ps and 9 ps for peak fluences of 1.5 ∙ *Φ*_thr_ and 3.0 ∙ *Φ*_thr_, respectively.

In air, the reflectivity decrease within the first tens of ps was previously attributed to decreasing surface density due to surface expansion^40^. For the ablation in water, an increasing reflectivity points to a reduced electron density and dilution of the generated plasma. Since the dilution occurs on a timescale of several ps, diffusion of electrons out of the plasma volume can be neglected^39^. However, since the collision of electrons with water molecules at the target/liquid interface occurs in the order of femtoseconds^41^, decomposition of water or trapping of electrons in local potential wells or solvated states may result in a decreased plasma density in water^42^.

### Domain 4, from 20 ps to 200 ps: spallation, phase explosion, and confinement of the ablation plume

**Fig. 3:**
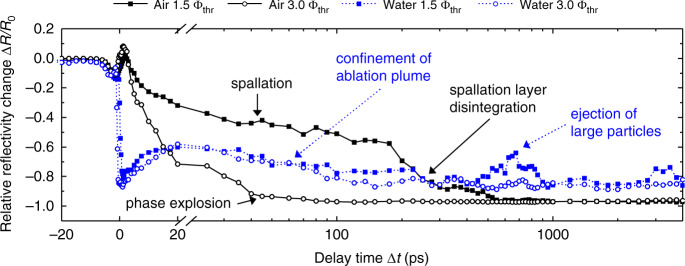
Relative reflectivity change in the spallation and phase explosion regime. Δ*R*/*R*_0_ is depicted for irradiation with peak fluences of 1.5 ∙ *Φ*_thr_ (full squares) and 3.0 ∙ *Φ*_thr_ (open circles) in air (black solid lines and symbols) and water (blue dotted lines and symbols)

To study the ablation dynamics in domain 4, the optical response is depicted at two fluences in Fig. 3 for delay times of up to 4 ns. For 1.5 ∙ *Φ*_thr_ similar transient behaviors are observed for air and water. Here, Δ*R*/*R*_0_ decreases with similar slopes between 20 and 200 ps. However, with an average value of Δ*R*/*R*_0_ = −0.5 the reflectivity in air remains above the average reflectivity of Δ*R*/*R*_0_ = −0.7 in water. When the sample is irradiated at 3.0 ∙ *Φ*_thr_, the reflectivity in air decreases to Δ*R*/*R*_0_ ≈ −0.97 when Δ*t* = 80 ps is exceeded; thus, the sample surface absorbs nearly all of the incident probe pulse. In the case of irradiation in water, a nonzero reflectivity with an average value of Δ*R*/*R*_0_ ≈ −0.75 is observed for 3.0 ∙ *Φ*_thr_.

As previously reported, the optical response upon irradiation above the ablation threshold fluence is strongly determined by the underlying ablation mechanism^43^. For the ablation of metal targets, the two predominant ablation mechanisms are photo-mechanical spallation and photo-thermal phase explosion^32^.

For photo-mechanical spallation to occur, the stress confinement condition *τ*_H_ ≤ *τ*_S_ ≈ *d*_eff_/*c*_S_ must be fulfilled^32^. More specifically, this means that the pulse energy deposition during the heating time *τ*_H_ must be finished before mechanical energy dissipation within the mechanical relaxation time *τ*_S_ occurs. Here, the heating time is given by the pulse duration *τ*_P_ or the electron-phonon equilibration time *τ*_e-ph_, whichever is longer. The mechanical relaxation time *τ*_S_ can be estimated by the ratio of the effective penetration depth *d*_eff_ = *d*_opt_ + *d*_diff_ and the speed of sound *c*_S_. For gold, the optical penetration depth *d*_opt_ at 1056 nm is approximately 12 nm (ref. ^25^) and the thermal diffusion length of hot electrons *d*_diff_ is approximately 100 nm (ref. ^44^). Consequently, with *c*_S_ = 2030 m s^−1^ (ref. ^45^), *τ*_S_ ≈ 55 ps exceeds *τ*_P_ = 3 ps and *τ*_e-ph_ = 20 ps (ref. ^46^). Thus the stress confinement condition is fulfilled for the experimental conditions employed in this work.

The prerequisite for the occurrence of photo-thermal phase explosion is the elevation of the lattice temperature to 0.9 ∙ *T*_C_, where *T*_C_ = (7400 ± 1100) K denotes the critical temperature of Au^47^. For an incident peak fluence of 1.5 ∙ *Φ*_thr_ the estimated maximum lattice temperatures of 3000 K– 4000 K are far below *T*_C_ (see Supplementary Information, Section 4, Supplementary Fig. S5) and spallation is the predominant ablation mechanism. At 3.0 ∙ *Φ*_thr_, the criterion for phase explosion is met since maximum lattice temperatures of 6300 and 8500 K are reached in air and water, respectively.

In the case of air, the reflectivity decreases with increasing peak fluence. This increase is attributed to the transition from spallation to phase explosion. In the former case, the material is ejected in the form of molten Au layers^17^ and Fresnel-like reflection at the air-Au interface results in a pronounced nonzero reflectivity. In the latter case, the ejected gas–liquid mixture no longer forms a sharp interface with the surrounding atmosphere. Here the gas–liquid mixture absorbs the probe pulse nearly completely^43^.

When the reflectivity in domain 4 (20–200 ps) is compared between water and air, it is observed that the reflectivity in water is below the reflectivity in air in the case of the spallation regime (*Φ*_0_ = 1.5 ∙ *Φ*_thr_). This lower reflectivity in water may be attributed to higher optical absorption above the spallation layer, originating from higher initial plasma densities (see time domain 2) and confinement of the plasma by the water layer^48^. In addition, a liquid spallation layer in air naturally exhibits a higher reflectivity than in water due to the higher refractive index mismatch between the liquid metal layer and air (see time domain 2). Furthermore, it is observed that the reflectivity in water decreases steadily in the fluence regime of spallation. It may be speculated that this reflectivity decrease originates from increased scattering and absorption due to surface roughening induced by Rayleigh–Taylor instabilities, which were predicted to occur on a similar timescale^17^. Another possible explanation for this steadily decreasing reflectivity may be an increasing plasma density and hence increasing plasma absorption due to a reduced trapping efficiency of electrons within the water layer as the water layer near the hot Au surface is rapidly brought to the supercritical state^16^. Compared to air, the reflectivity in water upon ablation in the phase-explosion regime (3.0 ∙ *Φ*_thr_) is increased significantly and nearly resembles the reflectivity dynamics observed in the spallation regime. This observation suggests that even though a highly absorbing gas–liquid mixture is ejected, the water layer confines the ablation plume, creating a sharp interface within the Au/water mixing region. Similar to ablation in the spallation regime, Fresnel-like reflection at this sharp interface results in a pronounced nonzero reflectivity.

### Domain 5, from 200 ps to 1 ns: spallation layer disintegration and NP generation

The fifth domain from Δ*t* = 200 ps to Δ*t* = 1 ns is highlighted by a crossing of the transient reflectivity curves in air and water for 1.5 ∙ *Φ*_thr_ (see Figs. 1 and 3). With Δ*R*/*R*_0_ steadily decreasing to −0.97, the reflectivity essentially vanishes in air after about 500 ps. For irradiation in water, the reflectivity shows a pronounced peak of Δ*R*/*R*_0_ = −0.66 at Δ*t* ≈ 650 ps. As depicted in Fig. 1a, this reflectivity peak is approximately limited to a region of *r* ≤ 2 µm around the irradiation center. At 3.0 ∙ *Φ*_thr_, the reflectivity in air remains at Δ*R*/*R*_0_ = −0.97, while in water, the peak at Δ*t* ≈ 700 ps with a value of Δ*R*/*R*_0_ = −0.82 is less pronounced compared to 1.5 ∙ *Φ*_thr_ (see Fig. 3). Since the reflectivity in air reaches the same values independent of the peak fluence, similar processes are likely to be the reason for the vanishing reflectivity within domain 5.

At a peak fluence of 3.0 ∙ *Φ*_thr_ the formation of a gas–liquid mixture induced by phase explosion has been identified as the reason for the vanishing reflectivity. Following this, the decreasing reflectivity at 1.5 ∙ *Φ*_thr_ may be identified as the disintegration of the molten spallation layer into liquid droplets, which would result in vanishing reflectivity since a sharp boundary between air and the ablation plume is no longer present^49^.

For ablation in water, the temporal occurrence of the reflectivity peak at 400 ps ≤ Δ*t* ≤ 900 ps matches the disintegration of the spallation layer in air and the onset of particle ejection predicted by computational methods for ablation of gold^17^ in water. Since the probe wavelength of 528 nm is located close to the Au surface plasmon resonance peak of ≈525 nm in water^8^, emerging Au NPs confined at the Au–water boundary would act like a mirror^50^, giving rise to an increased reflectivity. An explanation for the decreasing reflectivity for delay times exceeding 900 ps could be early cavitation bubble formation above the Au–water boundary, effectively shielding^11^ the probe pulse. Another possible explanation is the dispersion of the NPs within the water layer, leading to a reduced laser-NP interaction compared to closely packed NPs, where plasmon coupling results in an increased surface plasmon resonance intensity^51^.

### Domain 6, from 1 ns to 30 µs: pressure wave and cavitation bubble formation and propagation

Within domain six, which ranges from 1 ns to 30 µs, slower, but in the case of water, much more pronounced transient reflectivity dynamics are present compared to the previous domains (see Fig. 1). In the case of irradiation in air, the reflectivity essentially remains at zero up to delay times of 10 ns. Afterward, the reflectivity recovers slightly and eventually reaches its final state value of Δ*R*_inf_/*R*_0_ ≈ −0.9 at Δ*t* ≈ 100 ns. This increasing reflectivity is believed to be a consequence of the dilution of the particle cloud^49^, which is generated by the disintegration of the spallation layer (see domain 5).

**Fig. 4:**
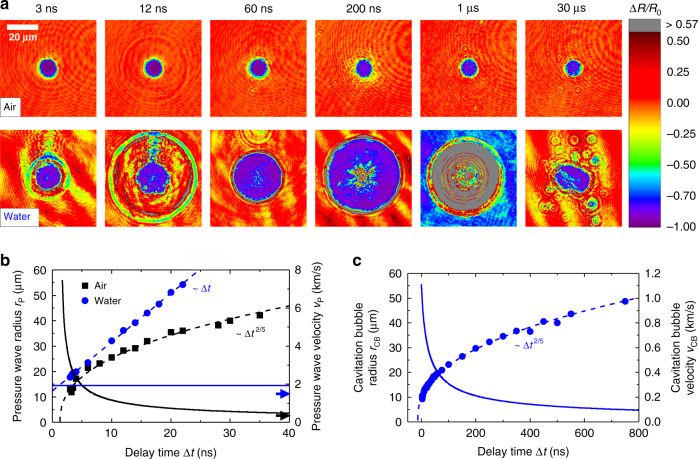
Pressure wave and cavitation bubble dynamics. **a** Color-coded reflectivity maps obtained after irradiation in air (top row) and water (bottom row) with pump pulses at a peak fluence of 1.5 ∙ *Φ*_thr_. Δ*R/R*_0_ = 0.57 corresponds to 100% reflectivity of the Au target in water. **b** Pressure wave radius *r*_P_ (dashed lines) and pressure wave velocity *v*_P_ (solid lines) as a function of the delay time Δ*t* in air (black lines and squares) and water (blue lines and circles). The speed of sound in air and water is indicated by black and blue arrows, respectively. **c** Cavitation bubble radius *r*_CB_ (blue circles and dashed line) and cavitation bubble velocity *v*_CB_ (blue solid line) as a function of Δ*t*. The velocities in (**b**), (**c**) were determined by the derivatives of the fitted radii

Irradiation in water causes the area of increased relative reflectivity to expand radially, starting at Δ*t* ≈ 200 ps (see Fig. 1a). Afterward, the reflectivity fluctuates between 1 ns and 400 ns in the irradiation center (see Fig. 1b). From Δ*t* = 400 ns, the reflectivity increases drastically and reaches a peak value of Δ*R*/*R*_0_ ≈ 24 at Δ*t* = 1 µs. To display such a high reflectivity increase, color coding above Δ*R*/*R*_0_ ≈ 0.57 was changed to gray in Figs. 1a and 4a. To further characterize the radial expansion of the reflectivity, Fig. 4a depicts the reflectivity maps for delay times ranging between 3 ns and 30 µs, which were obtained after irradiation of the Au target in air and water with peak fluences of 1.5 ∙ *Φ*_thr_.

In the case of air, a ring-shaped region of decreased reflectivity is observed at Δ*t* = 12 ns. During ablation in water, this region already appears at Δ*t* = 3 ns. The outwards propagating ring-shaped area of decreased reflectivity is induced by a pressure wave^14^. Figure 4b displays the pressure wave radius *r*_P_ and the pressure wave velocity *v*_P_ in air and water. In the case of air, the wave radius follows the dependency *r*_P_ ~ Δ*t*^2/5^, while for water a linear relationship between *r*_P_ and Δ*t* is obtained. This is consistent with previous observations^52^. In both cases, *v*_P_ exceeds the speed of sound of the respective medium thus the pressure waves propagate with supersonic velocities (*v*_P_ ≈ (0.4–6) km s^−1^ in air and *v*_P_ ≈ 2 km s^−1^ in water).

Following pressure wave propagation, a blurred area of decreased reflectivity appears within the vicinity of the irradiation region at Δ*t* = 60 ns in air. As Δ*t* increases, this area expands radially and is no longer visible at Δ*t* = 1 µs. The blurred region of decreased reflectivity surrounding the irradiation region in air was previously attributed to the outward propagating ablation plume^49^.

In contrast to ablation in air, the central region of vanishing reflectivity expands radially in water (see Figs. 1a and 4a) and covers the entire recorded image at Δ*t* = 300 ns (see Fig. 1a). The observed area of high absorption is associated with the cavitation bubble. Figure 4c depicts the cavitation bubble radius *r*_CB_ and the cavitation bubble velocity *v*_CB_, showing the agreement between the observed cavitation bubble dynamics and the predicted *r*_CB_ ~ Δ*t*^2/5^ dependence^14^. It should be noted that the model-fit of *r*_CB_ depicted in Fig. 4c predicts finite bubble radii at negative Δ*t*. However, this is unreasonable and attributed to deviations from the *r*_CB_ ~ Δ*t*^2/5^ dependence for Δ*t* < 10 ns. Furthermore, it is observed that the cavitation bubble is expanding with velocities below the speed of sound in water. At Δ*t* = 30 µs, the cavitation bubble collapses, and multiple microbubbles are observed. These microbubbles can persist up to milliseconds after pump-pulse impact^10^.

### Domain 7, from 30 µs to final state

At the longest investigated delay time of 30 µs, the reflectivity in air and water exhibits values that are comparable to the final state (Δ*R*_inf_/*R*_0_) of −0.89 ± 0.01 and −0.65 ± 0.04, respectively. The final state values were recorded 5 s after pump-pulse impact, therefore, transient dynamics can be excluded as the origin of the negative Δ*R*_inf_/*R*_0_. The negative Δ*R*_inf_/*R*_0_ may originate from increased absorption of the ablated region due to surface roughening^53^. This is in accordance with the observation that the ablation crater in air exhibits an increased surface roughness compared to water^27^, which is reflected in the increased Δ*R*_inf_/*R*_0_ after ablation in water, compared to ablation in air.

**Fig. 5:**
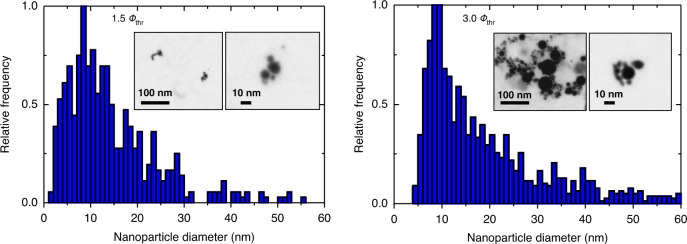
NP size distribution obtained after single-pulse irradiation in water with fluences of 1.5 ∙ *Φ*_thr_ (left panel) and 3.0 ∙ *Φ*_thr_ (right panel). TEM images of the generated NPs are provided as insets

The particle size distributions obtained after single-pulse ablation with peak fluences of 1.5 ∙ *Φ*_thr_ and 3.0 ∙ *Φ*_thr_ in water are depicted in Fig. 5. The TEM images reveal nearly spherical NPs with a wide distribution of diameters, peaking at ~10 nm, independent of the applied fluence. For irradiation with 1.5 ∙ *Φ*_thr_, the dispersion of the size distribution and the presence of larger particles is reduced compared to the 3.0 ∙ *Φ*_thr_ sample.

### Summary of the complete ablation process of Au in air and water

**Fig. 6:**
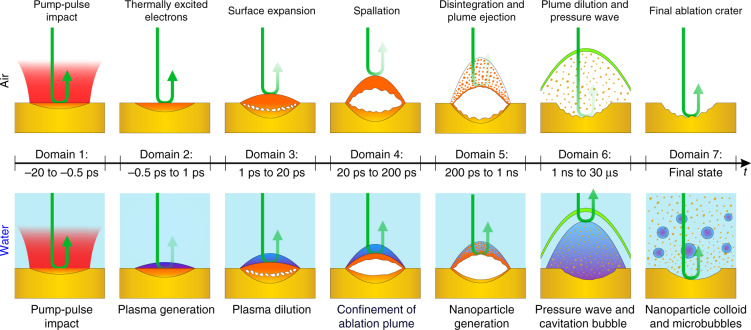
Schematic depiction of the ablation processes in the spallation regime (*Φ*_0_ = 1.5 ∙ *Φ*_thr_) within each delay time domain for air (top panels) and water (bottom panels). The incident pump-pulse is shown as a red area in domain 1. Yellow and orange areas depict solid and hot gold, respectively. The plasma, cavitation bubble, and microbubbles are colored purple. Pressure waves and the probe-pulse reflection are depicted in green and by green arrows, respectively. Note that this mechanistic sketch is not to scale, in particular, the domains 2–4 are two orders of magnitude laterally wider than high

Combining the results of the investigated temporal domains, a concise picture of the complete ablation process of Au in air and water is provided. Figure 6 schematically depicts the processes occurring within the investigated temporal domains.

In domain 1 (−20 ps to 0.5 ps), the laser pulse is absorbed by the conduction band electrons via inverse bremsstrahlung, and electron temperatures are elevated by several 10,000 K. The optical dynamics observed within this domain resemble each other in air and water and are a result of thermally excited electrons. Domain 2 (−0.5 ps to 1 ps) is characterized by a pronounced difference in the optical dynamics in air and water. In water, the electron injection from the Au surface into the water confinement layer and subsequent optical breakdown create a dense and highly absorbing plasma, which results in a sharp drop of the surface reflectivity. In air, such plasma is not observed and the optical dynamics are still governed by the thermal excitation of electrons. Surface expansion in air and decreasing plasma density in water determine the reflectivity dynamics in domain 3 (1–20 ps). The optical response in air within domain 4 (20–200 ps) is governed by the ablation mechanism. At a peak fluence of 1.5 ∙ *Φ*_thr_, photo-mechanical spallation induces propagation of a liquid layer, which results in the observation of finite reflectivity. If the laser peak fluence is increased to 3.0 ∙ *Φ*_thr_ photo-thermal phase explosion superimposes the spallation process and the ejection of a dense and highly absorbing gas–liquid mixture results in vanishing reflectivity after about 50 ps. When the irradiation is performed in water, the peak fluence has nearly no influence on the observed optical dynamics. Independent of the ablation mechanism (spallation at 1.5 ∙ *Φ*_thr_ and phase explosion at 3.0 ∙ *Φ*_thr_), the water layer confines the ablation plume, creating a Fresnel-like interface which exhibits a pronounced nonzero reflectivity. For air, the reflectivity remains approximately at zero for the phase-explosion regime in domain 5 (200 ps to 1 ns). In the spallation regime, the reflectivity vanishes after about 500 ps, reaching the same values as for irradiation with 3.0 ∙ *Φ*_thr_. This is a result of the disintegration of the spallation layer and generation of a highly absorbing ablation plume. In water, the emergence of large secondary NPs by hydrodynamic instability of the spallation layer is hypothesized within domain 5. This hypothesis is supported by the observation of a reflectivity peak, presumably due to the formation of a NP cloud mirror. Generation of supersonic pressure waves in air and water as well as the appearance of a cavitation bubble approximately 1 ns after pump-pulse impact characterize domain 6 (1 ns to 30 µs). Furthermore, the bubble collapse at delay times ranging between 20 and 30 µs is observed here. The final state reflectivity in domain 7 exhibits a higher value in water compared to air. This is attributed to the lower surface roughness of the final ablation crater in water. Here the irradiated region is in equilibrium with its environment. In the case of water, primary NPs with diameters around 10 nm and secondary NPs with diameters around 40 nm are in solution. Furthermore, persistent microbubbles are observed in the solution after the laser ablation process has finished.

It may be speculated that the conditions for small primary NP generation are already established within domain 4 (20–200 ps). Here the generation of Au vapor by spallation or phase explosion may lay the foundation for condensation of the primary NP fraction. Combined with the observation of the ejection of large secondary NPs in domain 5 (200 ps to 1 ns), this may indicate the two mechanisms of primary and secondary NP generation, which are believed to be responsible for the bimodal size distribution frequently observed in ultrashort LAL^16^. Since it is speculated that the particle generation occurs several 100 ps after pulse impact, the electron injection occurring a few ps after pulse impact within domain 2 is believed to have no immediate influence on the particle generation mechanisms. However, the generated highly absorbing plasma may partly shield the pump-pulse and is thus a key process determining the absorption within the initial stage of the LAL process. Therefore, the early electron injection must be considered in computational studies to allow a quantitative comparison of simulation results and experiments.

Finally, the experimental results may explain the pulse duration dependency of the NP productivity. Dittrich et al. observed that the power-specific productivity during LAL is low for ultrashort pulses of 3 ps (12 mg W^−1^ h^−1^)) and for pulses of 5 ns (7.5 mg W^−1^ h^−1^), while a pulse duration of 1 ns yields optimum productivity (43 mg W^−1^ h^−1^)^24^. The lower specific productivity for ps pulses in water may be attributed to the electron injection and plasma formation in domain 2. Since the highly absorbing plasma is generated above the surface (10–100 nm) after 0.5 ps already within the laser pulse duration (3 ps), the trailing edge of the laser pulse may be absorbed above the surface. Hence, only a fraction of the pulse energy is available for the ablation process, reducing the specific productivity. In the case of longer pulses (ns), plasma shielding may be absent since lower electron temperatures lead to a decreased electron-emission rate and hence the initial electron density is not reached here. However, the cavitation bubble emerging at approximately 1 ns after pulse impact may scatter and shield a significant portion of the pulse energy, resulting in a lower productivity for 5 ns pulses. Thus, the high productivity reported at a pulse duration of about 1 ns may be achieved by avoiding plasma shielding and depositing the pulse energy before the cavitation bubble emerges^54^.

## Discussion

In summary, the complete spatiotemporal picosecond laser-induced ablation dynamics of Au immersed in air and water for various peak fluences have been investigated from the picosecond to the microsecond timescale. PPM experiments provide transient reflectivity over six orders of magnitude, ranging from ps to µs. The water layer is found to significantly influence the ablation dynamics over the entire investigated timescale. The water layer facilitates plasma generation due to electron-emission-induced optical breakdown, confines the ablation plume on a timescale of several hundred ps, and promotes cavitation bubble generation after ~1 ns. In addition, the initial stage of primary nanoparticle generation is speculated to occur after about 100 ps, when Au vapor is generated by spallation or phase explosion. It is furthermore demonstrated that a narrow temporal window ranging from ~200 ps to 1 ns exists, within which the generation of large secondary nanoparticles is speculated, confirming the computational prediction of secondary NP generation within this temporal range.

The experimental findings present a complete view of the mechanisms influencing LAL from the early stage during the first picoseconds until the cavitation bubble collapses and the nanoparticles are ejected. The experimental observations of the LAL process are in good agreement with established computational predictions providing a clear picture of the processes involved in LAL. A deep understanding of the overall LAL dynamics is fundamental to open up the door to further optimize the process towards an enhanced control of the generated nanoparticles size distribution and composition or increased production rates. This in turn enables the optimization of applications in fields such as plasmonics, catalysis, biomedicine, and 3D printing, which opens the field of LAL to a broad community.

## Materials and methods

### Sample preparation and characterization

Polycrystalline Au samples with a purity of 99.99% and a thickness of 1 mm were embedded in an epoxy resin matrix. The samples were sanded and subsequently polished with 9, 3, and 1 µm polycrystalline diamond suspensions. In a final step, an attack polish consisting of a mixture of 50 nm Al_2_O_3_ NPs and a few mL KI/I_2_ solution was performed. The resulting average surface roughness of *R*_a_ = 10 nm was determined with a confocal microscope (Leitz, Ergoplan).

### Ultrafast pump-probe microscopy

The spatiotemporal relative reflectivity transients were recorded using a pump-probe microscopy setup. For a schematic depiction of the setup, see Supplementary Information, Section 1, Supplementary Fig. S1. Pulses with a wavelength of 1056 nm and a full width at half maximum (FWHM) pulse duration of 530 fs were generated by a Nd:Glass laser (HighQLaser, femtoREGEN). The pulses were split into a pump- and a probe-branch employing a half-wave-plate (HWP) polarizing beam splitter (PBS) combination. Before a mechanical shutter (MS) extracted single pump pulses from the pulse train, the pump-pulse energy was adjusted with another HWP-PBS combination. A photodiode (PD) monitored the pump-pulse energy. Afterward, the FWHM pump-pulse duration was increased to 3 ps with a pulse stretcher. The p-polarized pump pulses were focused onto the sample using a lens with a focal length of *f* = 100 mm. The resulting Gaussian intensity distribution in the focal plane was characterized under normal incidence with a focal beam profiler (PRIMES GmbH, MicroSpotMonitor), yielding a beam waist radius of *w*_0_ = (15 ± 1) µm at 1/*e*^2^ intensity level. Before each experiment the incident pump-pulse energy was measured by a power meter (Coherent Inc., PS10Q). In air the pump-pulse was incident under an angle of 35° on the sample. For laser ablation in liquids, the sample was covered with a 4-mm thick layer of deionized water. This specific thickness has been chosen to avoid surface waves and possible break-up of the water surface by the expanding cavitation bubble^55^. Here the incidence angle decreased to 25.6° due to refraction at the air/water interface. The oblique incidence in air and water resulted in an elliptical beam profile. Independent of the immersion medium, the minor beam waist radius *w*_min_ was equal to *w*_0_ measured under normal incidence, while the major beam waist radii *w*_maj_ were 18.3 µm and 16.6 µm for air and water, respectively.

The probe pulse was frequency-doubled to spectrally separate it from the pump-pulse and thus suppress scattered pump radiation within the imaging system. This resulted in a wavelength of 528 nm and a pulse duration of 500 fs. Afterward an optical delay line introduced a variable delay time Δ*t* of up to 4 ns between the pump- and probe pulse. For delay times exceeding 4 ns, a second electronically triggered laser source with a wavelength of 532 nm and a pulse duration of 600 ps was used (InnoLas Laser GmbH, picolo AOT). The probe pulses were then coupled into the microscopy section of the setup. Illumination of the sample surface through a long working distance microscope objective (MO) (×50, NA = 0.42) was performed under normal incidence. The reflected portion of the probe pulse was collected by the same MO and imaged onto a charge-coupled device camera (CCD) (PCO AG, pco.pixelfly usb) with a tube lens. A band-pass filter (BPF), centered at (530 ± 10) nm was located in front of the camera to block undesired pump- as well as plasma radiation. The setup provides lateral and temporal resolutions of ~630 nm and 500 fs, respectively. Temporal synchronization of the MS, PD, CCD, and picosecond laser was achieved by a delay generator (Stanford Research Systems, DG645).

The delay time zero point Δ*t* = 0 ps of the system is defined as the maximum overlap of the pump- and probe pulse, i.e., when the peaks of both pulses overlap. This was calibrated in air and water by measuring the instantaneous reflectivity response of silicon to sub-threshold pump pulses^56^, providing an accuracy of ~±100 fs.

For each Δ*t*, the sample was translated to illuminate a pristine area, where a sequence of three images was acquired. First, a reference image (*R*_0_) was taken 5 s before pump-pulse impact. Afterward, the images (*R*(Δ*t*)) at the desired delay time Δ*t* and (*R*_inf_) 5 s after pump-pulse impact were recorded. Finally, the transient relative reflectivity change Δ*R/R*_0_ = (Δ*R* *−* *R*_0_)*/R*_0_ and the final state relative reflectivity change Δ*R*_inf_*/R*_0_ = (Δ*R*_inf_ − *R*_0_)*/R*_0_ were calculated for each pixel.

### Single-pulse laser ablation for colloid production

The single-pulse ablation experiments for colloid production were performed by employing a ps laser source (Ekspla Co., Atlantic series, 1064 nm, 100 kHz, and 10 ps). The focal spot size is adjusted to match the value employed in the pump-probe experiments (*w*_0_ = (15 ± 1) µm at 1/*e*^2^ intensity level). In order to be able to perform a statistically relevant analysis of the size distribution by scanning transmission electron microscopy (STEM), the NP concentration was increased by setting the number of single-pulse irradiation events to 3600. The laser source was synchronized with a galvanometric scanner and a delay time of 5 s was applied between each of the single-pulse irradiation events. During this time, the scanning system displaced the beam so each single-pulse irradiated an unprocessed area of the sample, ensuring that incubation, heat accumulation and shielding effects were neglectable. The generated colloidal gold NPs were deposited on carbon-coated copper grids and dried for scanning transmission electron microscopy (ThermoFisher Scientific Inc., ESEM Quanta 400 FEG) characterization. A standard protocol was adopted for the STEM samples preparation; three drops of the colloid were dropped cast on the grids for the 3.0 ∙ *Φ*_thr_ samples and six for the 1.5 ∙ *Φ*_thr_ to compensate for the concentration difference and in order to characterize a statistically relevant amount of nanoparticles for both experimental conditions. The acquired images are computer analyzed to obtain the nanoparticle size distributions using the ParticleSizer plugin in ImageJ software.

## Supplementary information

Supplementary Information
