# Supplementary material for: Comparison of ultrashort pulse ablation of gold in air and water by time-resolved experiments
Source: Light Sci Appl. 2022 Mar 23;11:68. doi: 10.1038/s41377-022-00751-6 (PMC8943017; doi:10.1038/s41377-022-00751-6)
Supplement: Supplementary file 1 — Supplementary Information [file 41377_2022_751_MOESM1_ESM.docx]

**Supplementary Information for**

Comparison of ultrashort pulse ablation of gold in air and water by time-resolved experiments

Maximilian Spellauge^1,2^, Carlos Doñate-Buendía^2,3^, Stephan Barcikowski^2^, Bilal Gökce^2,3,*^, Heinz P. Huber^1,*^

^1^Department of Applied Sciences and Mechatronics, Munich University of Applied Sciences, Lothstraße 34, 80335 Munich, Germany

^2^Technical Chemistry I and Center for Nanointegration Duisburg-Essen (CENIDE), University of Duisburg-Essen, 45141 Essen, Germany

^3^Materials Science and Additive Manufacturing, School of Mechanical Engineering and Safety Engineering, University of Wuppertal, 42119 Wuppertal, Germany

^*^Corresponding authors: [goekce@uni-wuppertal.de](mailto:goekce@uni-wuppertal.de) (Bilal Gökce), [heinz.huber@hm.edu](mailto:heinz.huber@hm.edu) (Heinz P. Huber)

**1. Experimental Setups**

Fig. S1 displays the pump-probe microscopy setup.


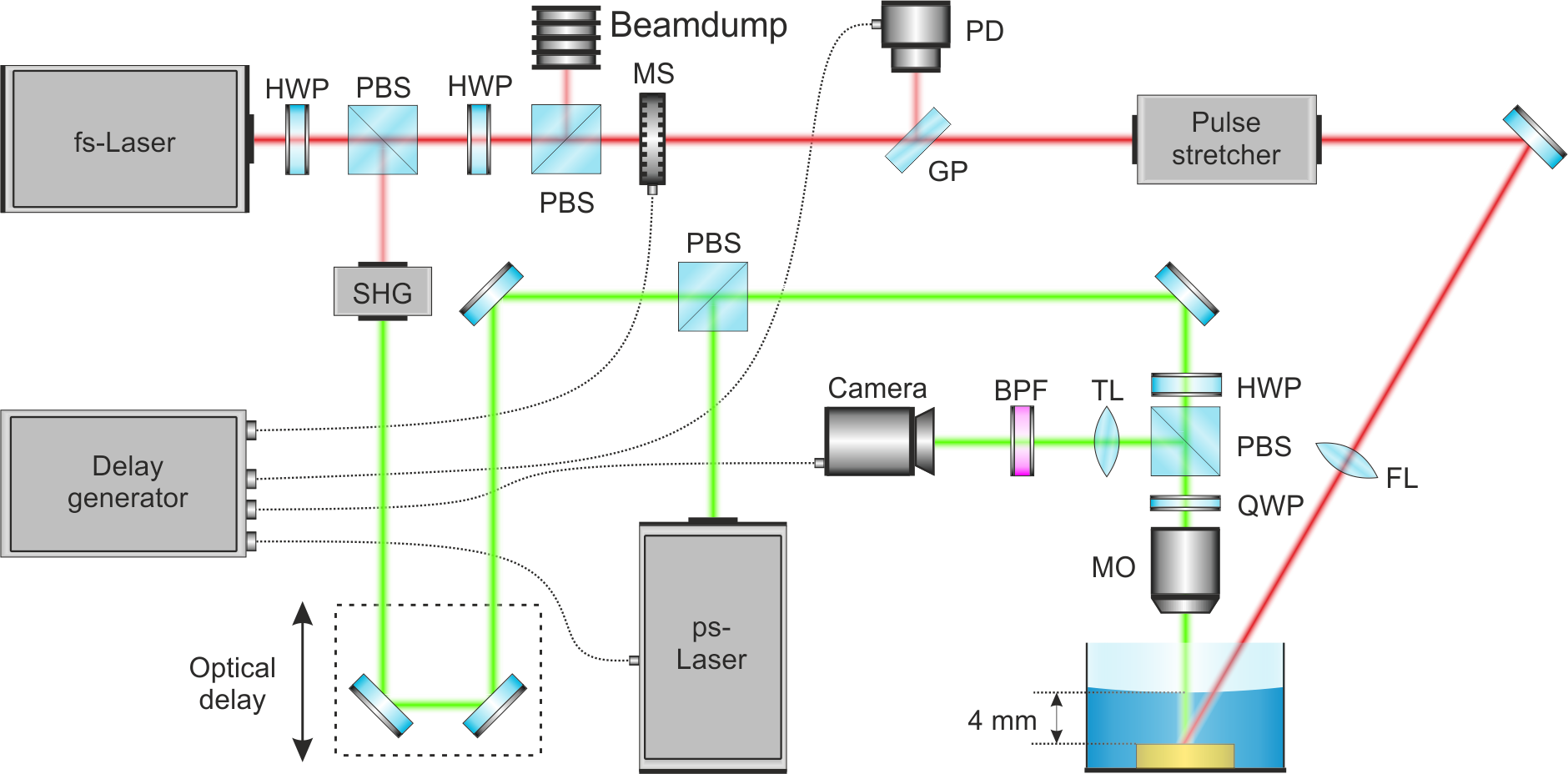


**Fig. S1** Schematic depiction of the pump-probe microscopy setup. HWP: half-wave plate, PBS: polarizing beamsplitter, MS: mechanical Shutter, GP: glass plate, PD: photodiode, FL: focusing lens (plano-convex, focal length of 100 mm), SHG: second-harmonic generation unit, QWP: quarter-wave plate, MO: microscope objective (50x, NA = 0.42) TL: tube lens, BPF: band-pass filter ((530 ± 10) nm).

The pump-probe microscope allows for the investigation of the transient reflectivity with an accuracy of approximately 0.5 % and a temporal resolution of the respective probe-pulse duration of about 500 fs for delay times Δ*t* ≤ 4 ns and of 600 ps for Δ*t* ≥ 4 ns. A detailed description of the setup can be found in the main manuscript.

**2. Single-pulse ablation threshold fluence**

In order to study the influence of the ambient medium on the ablation energetics, the ablation threshold fluence in air and water have been determined.

According to the *D*^2^-model, the following equation can be derived under the assumption of a Gaussian spatial intensity distribution and the existence of an ablation threshold fluence *Φ*_thr_ which must be exceeded for ablation to occur^1^:

$D^{2}=2\cdot w_{0}^{2}\cdot\ln\left(\frac{\Phi_{0}}{\Phi_{\text{thr}}}\right)$ S1

Here *D* denotes the ablation crater diameter, *w*_0_ the beam waist radius at *e*^-2^ intensity level, and *Φ*_0_ the peak fluence i.e. the peak surface energy density. *Φ*_0_ in dependence of the pulse energy *E*_P_ and the incidence angle *ϑ* is given by the following equation^2^:

$\Phi_{0}=\frac{2\cdot E_{\text{P}}\cdot\cos(\vartheta)}{\pi\cdot w_{0}^{2}}$ S2

Under oblique incidence, the resulting elliptical spatial intensity distribution on the sample surface is described by a the minor beam waist radius *w*_min_ = *w*_0_ and the major beam waist radius *w*_maj_ = *w*_0_/cos(*ϑ*). Figure S2a displays *D*^2^, measured along the minor axis of the ablation crater in air (black solid squares) and in water (blue solid circles) as a function of the incident *Φ*_0_. Black and blue solid lines depict the fit of equation S1 to the measured data for air and water, respectively.


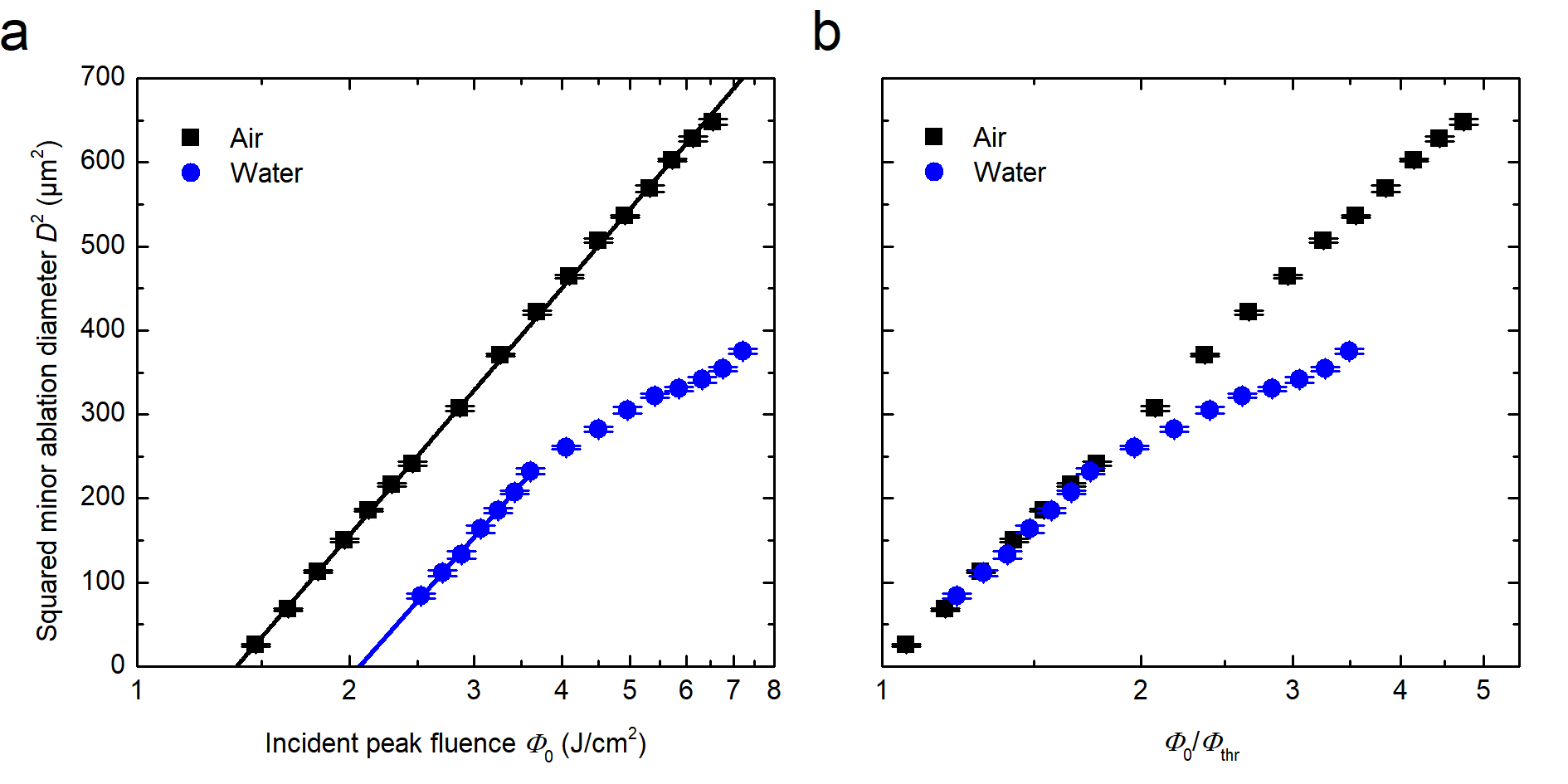


**Fig. S2** (a) Experimental determined squared minor ablation diameter *D*^2^ as a function of the incident peak fluence *Φ*_0_ in air (black solid squares) and water (blue solid circles). The fit of equation S1 to the measured data is indicated by black and blue solid lines for air and water, respectively. (b) *D*^2^ in dependence of *Φ*_0_, normalized by the respective ablation threshold fluence *Φ*_thr_.

In the case of air, the whole presented data range is used for the fitting procedure, while in case of water the fitting procedure was limited to the data points up to 3.6 J cm^-2^. Table S1 summarizes the results of the fitting procedure.

**Table S1** Fitted ablation threshold fluence *Φ*_thr_ and minor beam waist radius *w*_0_.

|  | *Φ*_thr_ (J cm^-2^) | *w*_0_ (µm) |
| --- | --- | --- |
| Air | 1.4 ± 0.1 | 14.6 ± 0.1 |
| Water | 2.1 ± 0.1 | 14.4 ±0.1 |

The squared minor ablation diameter *D*^2^ in dependence of *Φ*_0_, normalized by the respective ablation threshold fluence *Φ*_thr_ is depicted in figure S2b.

The threshold fluence in water is signicantly larger compared to the threshold fluence in air. By normalizing the peak fluence to the ablation threshold fluence, similar ablation diameters are obtained up to *Φ*_0_ = 1.8∙*Φ*_thr_.

**3. Optical breakdown threshold fluence**

The optical breakdown threshold in water for the employed experimental parameters was calculated to investigate if an optical breakdown may occur during the laser ablation experiments.

In the case of water, where the free electron density is negligible, two threshold intensities are associated with optical breakdown for ps pulses^3^. The first threshold intensity *I*_m_ is the intensity needed in order to generate an initial free electron density by multiphoton ionization:

$I_{\text{m}}=\frac{1}{B}\cdot\left(\frac{\rho_{0,\min}}{A\cdot0.1\cdot\tau_{\text{P}}}\right)^{\frac{1}{K}}$ S3

Here *τ*_P_ is the full width at half maximum laser pulse duration, *K* = 6 the number of photons needed for ionization. *A*= 1.3908∙10^40^ s^-1^∙m^-3^ and *B* = 4.9231∙10^-18^ m^2^∙W^-1^ are constants for water at a laser wavelength of *λ* = 1064 nm (ref. 4). The minimum value of the initial free electron density is calculated by *ρ*_0,min_ = *N*_0_/*V*_f_, where *N*_0_ is the minimum number of free electrons within the focal volume *V*_f_ = π^2^∙w_0_^4^/λ. The second threshold intensity *I*_C_ is the intensity needed in order to sustain a cascade ionization process:

$I_{\text{C}}=\left(C\cdot\frac{1+\omega^{2}\cdot\tau^{2}}{\tau}\right)\cdot\left[g+\frac{2}{\tau_{\text{P}}}\cdot\ln\left(\frac{\rho_{\text{cr}}}{\rho_{0,\min}}\right)\right]+C\cdot\frac{m\cdot\omega^{2}}{M}$ S4

with

$C=\frac{m\cdot c\cdot n_{0}\cdot\varepsilon_{0}\cdot E_{\text{ion}}}{e^{2}}$ S5

In the equations above *m*, *e*, *c*, *ε*_0_, ω and M are the electron mass, elementary charge, speed of light in vacuum, vacuum permittivity, laser angular frequency and mass of a water molecule, respectively. Furthermore, *ρ*_cr_ = 10^21^ cm^-3^ is the critical plasma density for fs and ps pulses^5^, *τ* = 10^15^ s the electron colission time^6^, *n*_0_ = 1.32 the refractivive index of water at 1056 nm (ref. 7) and *E*_ion_ = 6.5 eV the ionization energy of water^8^. Since the electron loss rate *g* can be neglected for pulse durations smaller 10^-8^ s, *g* was set to 0 in the calculations for a pulse duration of 3 ps (ref. 3). If *I*_C_ < *I*_M_, then *I*_M_ is the optical breakdown threshold intensity. However, if *I*_C_ > *I*_M_ then the optical breakdown threshold is located at the intersecion of the two *ρ*_0,min_ dependent threshold values.
In order to allow for a comparison of the calculated breakdown threshold intensities with peak fluences investigated in this study, the intensities *I* are converted to fluences *Φ* by *Φ* ≈ *I*∙*τ*_P_/0.94, where *τ*_P_ is the full width at half maximum pulse duration and the factor of 0.94 results from the temporal Gaussian intensity distribution. Figure S3 depicts the multiphoton ionization threshold fluence *Φ*_M_ (blue solid line) and the cascade ionization threshold fluence *Φ*_C_ (blue dotted line) as a function of the initial free electron density *ρ*_0,min_.


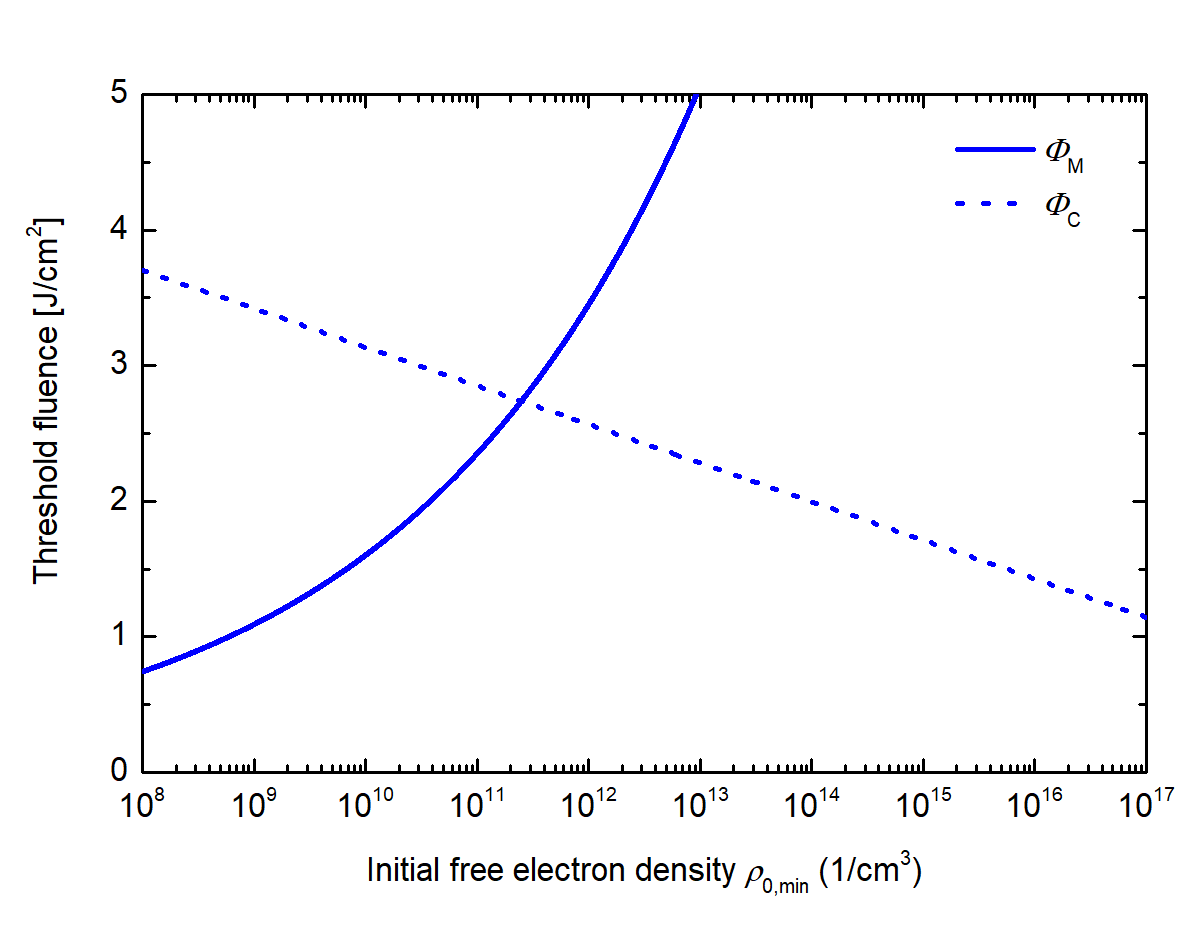


**Fig. S3** Calculated multiphoton initiation threshold fluence *Φ*_M_ (blue solid line) and cascade ionization threshold fluence *Φ*_C_ (blue dotted line) as a function of the initial free electron density *ρ*_0,min_.

The intersection of *Φ*_M_ and *Φ*_C_ in figure S3 yields an optical breakdown threshold fluence of *Φ*_OB_ ≈ 2.75 J cm^-2^. This value corresponds to an incident peak fluence of 1.7∙*Φ*_thr_.
To obtain the position within the liquid layer, where *Φ*_OB_ is exceeded, the peak fluence *Φ*_o_ in dependence of the distance *z* to the focal plane is calculated by:

$\Phi_{0}(z)=(1-R_{\text{AW}})\cdot\exp\left(-\frac{z+z_{0}}{d_{\text{opt}}}\right)\cdot\frac{2\cdot E_{\text{P}}\cdot\cos(\vartheta)}{\pi}\cdot\left(w_{0}^{2}\cdot\left(1+\left(\frac{\lambda\cdot z}{\pi\cdot w_{0}^{2}}\right)\right)\right)^{-1}$ S6

Here the first term on the right side of the equation represents the reflection losses *R*_AW_ at the air-water interface. The second term describes the linear absorption losses within the pulse propagation path *z*_0_ in water, which is caracterized by the optical penetration depth *d*_opt_. The dependence of the peak fluence on *w*_0_(*z*) is given by the last two terms of the equation.
By using *R*_AW_ = 0.01 and *d*_opt_ = 17.3 mm for water^7^, the curves shown in figure S4 are obtained for different multiples of the incident ablation threshold fluence. The air-water boundary and the focal plane are located at *z* = -4.3 mm and *z* = 0 mm, respectively.


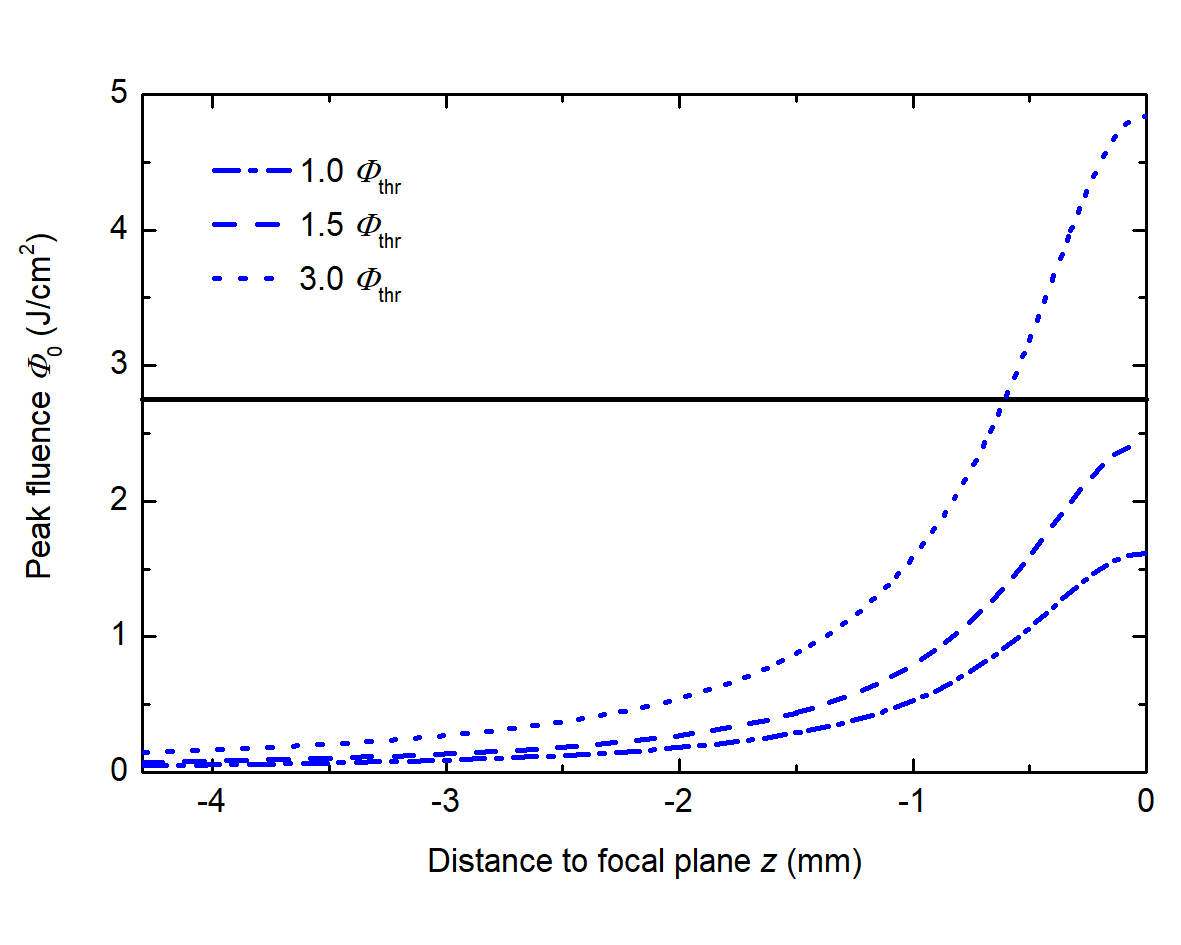


**Fig. S4** Peak fluence *Φ*_o_ in dependence of the distance *z* to the focal plane for 1.0*∙Φ*_thr_ (blue dash-dotted line), 1.5*∙Φ*_thr_ (blue dashed line) and 3.0*∙Φ*_thr_ (blue dotted line). The optical breakdown threshold fluence is indicated by a black solid line.

The calculations show that the optical breakdown threshold fluence is only exceeded for the highest employed peak fluence of *Φ*_0_ = 3.0∙*Φ*_thr_.

**4. Two-temperature model**

The temperatures of the electronic and lattice sub-system were calculated in order to allow for an estimation of the thermionic emission yield and the occurance of phase explosion, respectively.

The electron temperature *T*_e_ and the lattice temperature *T*_i_ of gold following laser irradiation are estimated based on the one-dimensional (1D) two-temperature model (TTM). The two coupled differential equation of the 1D-TTM are^9^:

$\begin{matrix} c_{\text{e}}\cdot\frac{\partial T_{\text{e}}}{\partial t}=\frac{\partial}{\partial z}\left(k_{\text{e}}\cdot\frac{\partial T_{\text{e}}}{\partial z}\right)-g\cdot(T_{\text{e}}-T_{\text{i}})+S \\ c_{\text{i}}\cdot\frac{\partial T_{\text{i}}}{\partial t}=g\cdot(T_{\text{e}}-T_{\text{i}}) \end{matrix}$ S7

Here *c*_e_ denotes the electron heat capacity, *c*_i_ = 2.45∙10^6^ J m^-3^ K^-1^ the lattice heat capacity^10^, *k*_e_ the electron thermal conductivity, *g* the electron-phonon coupling constant, and *S* the laser source term. The *T*_e_ dependence of *c*_e_ and *g* for electron temperatures up to 50 kK was accounted for^11^. The dependence of the electron thermal conductivity on *T*_e_ and *T*_i_ was estimated by^12^:

$k_{\text{e}}(T_{\text{e}},T_{_{\text{i}}})=353\frac{\text{W}}{\text{mK}}\cdot\frac{(\vartheta_{\text{e}}^{2}+0.16)^{5/4}\cdot(\vartheta_{\text{e}}^{2}+0.44)\cdot\vartheta_{\text{e}}}{(\vartheta_{\text{e}}^{2}+0.092)^{1/2}\cdot(\vartheta_{\text{e}}^{2}+0.16\cdot\vartheta_{\text{i}})}$ S8

where *ϑ*_e_ = *k*_b_*T*_e_/*E*_F_, *ϑ*_i_ = *k*_b_*T*_i_/*E*_F_ and *E*_F_ the fermi energy. The laser source term *S* in dependence of the time *t* and the depth *z* is given by:

$S(z,t)=\frac{1.76\cdot A\cdot\Phi_{0}}{2\cdot\tau_{\text{P}}\cdot d_{\text{opt}}}\cdot\exp\left(-\frac{z}{d_{\text{opt}}}\right)\cdot\text{sech}^{2}\left(\frac{1.76\cdot t}{\tau_{\text{P}}}\right)$ S9

Here *A* denotes the absorption of Au at 1056 nm pump-wavelength^13^. In the case of Au in water, reflection losses at the air-water interface as well as linear absorption losses within the water volume have been taken into account^7^. The resulting *T*_e_ (top panels) and *T*_i_ (bottom panels) are depicted in figure S5 for irradiation in air (left panels) and water (right panels) for peak fluences of 1.0*∙Φ*_thr_ (dash-dotted line), 1.5*∙Φ*_thr_ (dashed line), and 3.0*∙Φ*_thr_ (dotted line). The maximum *T*_e_ and *T*_i_ are summarized in table S2.


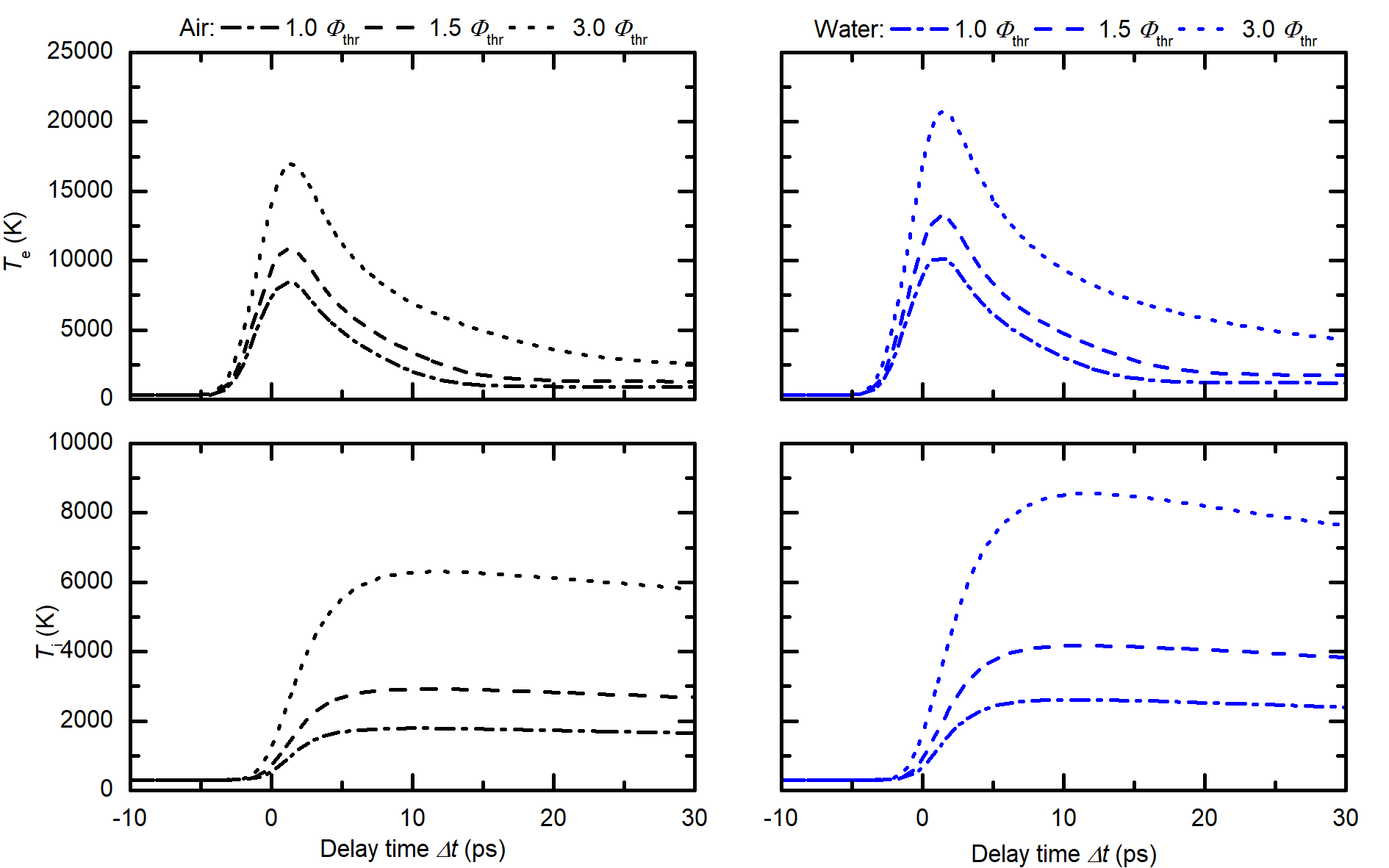


**Fig. S5** Electron temperatures *T*_e_ (top panels) and lattice temperatures *T*_i_ (bottom panels) for irradiation of Au in air (left panels, black lines) and water (right panels, blue lines) for peak fluences of 1.0*∙Φ*_thr_ (dash-dotted line), 1.5*∙Φ*_thr_ (dashed line) and 3.0*∙Φ*_thr_ (dotted line).

**Table S2** Maximum electron temperatures *T*_e,max_ and ion temperatures *T*_i,max_. at the gold surface

|  | 1.0*∙Φ*_thr_ | | 1.5*∙Φ*_thr_ | | 3.0*∙Φ*_thr_ | |
| --- | --- | --- | --- | --- | --- | --- |
|  | Au/Air | Au/Water | Au/Air | Au/Water | Au/Air | Au/Water |
| *T*_e,max_ (K) | 8448 | 10264 | 10851 | 13216 | 16935 | 20725 |
| *T*_i,max_ (K) | 1791 | 2615 | 2919 | 4171 | 6304 | 8557 |

Note that the heat flux from the Au surface to the water layer, phase-transitions, fluence dependent absorption of the Au target and non-linear absorption within the water volume have been neglected in the 1D-TTM calculations. Thus the temperatures presented here are a rough estimation.

**5. Thermionic emission**

The electron temperatures, which were estimated with the TTM in section 4 allow for an estimation of the thermionic electron emission yield.

The total thermionic electron yield *N*_esc_ in the space-charge limited regime can be estimated by the following equation^14^ (CGS units):

$N_{\text{esc}}=\frac{k_{\text{B}}T_{\text{peak}}R_{1}}{ae^{2}}\cdot\log\left[1+C\tau_{\text{P}}\pi R_{2}ae^{2}k_{\text{B}}T_{\text{peak}}\cdot\exp\left(-\frac{E_{\text{f}}-\mu+W}{k_{\text{B}}T_{\text{peak}}}\right)\right]$ S10

Here *k*_B_ is the Boltzmann constant, *T*_peak_ the peak electron temperature, *R*_1_ ≈ 0.59∙*w*_0,maj_ and *R*_2_ ≈ 0.59∙*w*_0,min_ the half width at half maximum radii of the eliptical laser spot, *τ*_P_ the laser pulse duration, *E*_f_ the Fermi energy, *µ* the chemical potential and *W* the work function. The constant *a*= 16/(3*π*) for a uniform disk and *C* = 4π*m*/*h*^3^, where *m* is the electron mass and *h* is the Planck constant.
The electron density above the sample material is then given by:

$$n_{\text{esc}}=\frac{N_{\text{esc}}}{\pi\cdot R_{1}\cdot R_{2}\cdot d_{e}}$$

Where *d*_e_ is the electron penetration depth, which ranges between 10 nm and 100 nm in water. By this, the minimum and maximum thermionic emission electron densities are calculated and displayed in Figure S6 as a function of the normalized peak fluence.


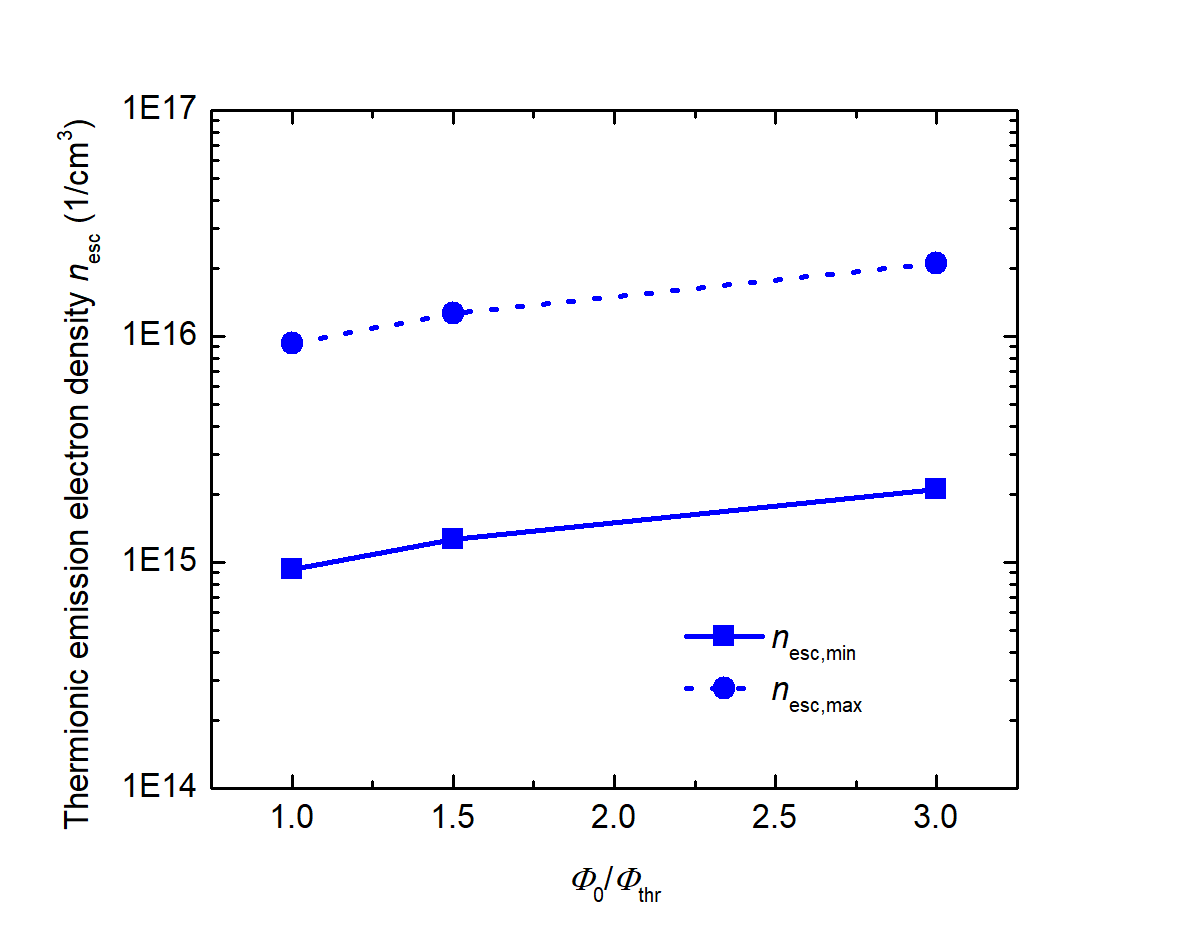


**Fig. S6** Minimum and maximum thermionic emission electron density as a function of the threshold fluence normalized peak fluence.

It is observed that the thermionic electron emission density does not change significantly with increasing mulitiple of the threshold fluence.

**References**

1. Liu, J. M. Simple technique for measurements of pulsed Gaussian-beam spot sizes. *Opt. Lett.* **7**, 196 (1982).

2. Boerner, P., Hajri, M., Wahl, T., Weixler, J. & Wegener, K. Picosecond pulsed laser ablation of dielectric rods: Angle-dependent ablation process model for laser micromachining. *J. Appl. Phys.* **125**, 234902 (2019).

3. Kennedy, P. K. A First-Order Model for Computation of Laser-Induced Breakdown Thresholds in Ocular and Aqueous Media: Part I—Theory. *IEEE J. Quantum Electron.* **31**, 2241–2249 (1995).

4. Vogel, A., Nahen, K., Theisen, D. & Noack, J. Plasma formation in water by picosecond and nanosecond Nd:YAG laser pulses. I. Optical breakdown at threshold and superthreshold irradiance. *IEEE J. Sel. Top. Quantum Electron.* **2**, 847–860 (1996).

5. Noack, J. & Vogel, A. Laser-induced plasma formation in water at nanosecond to femtosecond time scales: calculation of thresholds, absorption coefficients, and energy density. *IEEE J. Quantum Electron.* **35**, 1156–1167 (1999).

6. Bloembergen, N. Laser-induced electric breakdown in solids. *IEEE J. Quantum Electron.* **10**, 375–386 (1974).

7. Hale, G. M. & Querry, M. R. Optical Constants of Water in the 200-nm to 200-μm Wavelength Region. *Appl. Opt.* **12**, 555 (1973).

8. Sacchi, C. A. Laser-induced electric breakdown in water. *J. Opt. Soc. Am. B* **8**, 337 (1991).

9. Anisimov, S. I., Kapeliovich, B. L. & Perel-man, T. L. Electron emission from metal surfaces exposed to ultrashort laser pulses. *J. Exp. Theor. Phys.* **66**, 375–377 (1974).

10. Block, A. *et al.* Tracking ultrafast hot-electron diffusion in space and time by ultrafast thermomodulation microscopy. *Sci. Adv.* **5**, eaav8965 (2019).

11. Lin, Z. & Zhigilei, L. V. Thermal excitation of d band electrons in Au: implications for laser-induced phase transformations. in *High-Power Laser Ablation VI* (ed. Phipps, C. R.) vol. 6261 62610U (2006).

12. Anisimov, S. I. & Rethfeld, B. Theory of ultrashort laser pulse interaction with a metal. in *Proc. of SPIE* (eds. Konov, V. I. & Libenson, M. N.) 192–203 (1997). doi:10.1117/12.271674.

13. Johnson, P. B. & Christy, R. W. Optical Constants of the Noble Metals. *Phys. Rev. B* **6**, 4370–4379 (1972).

14. Riffe, D. M. *et al.* Femtosecond thermionic emission from metals in the space-charge-limited regime. *J. Opt. Soc. Am. B* **10**, 1424 (1993).
